# Remnant cholesterol and the risk of aortic valve calcium progression: insights from the MESA study

**DOI:** 10.1186/s12933-023-02081-2

**Published:** 2024-01-09

**Authors:** Ze-Hua Li, Qing-Yun Hao, Yu-Hong Zeng, Jing-Bin Guo, Shi-Chao Li, Jing-Wei Gao, Ping-Zhen Yang

**Affiliations:** 1 Department of Cardiology, Laboratory of Heart Center, Zhujiang Hospital, Southern Medical University, Guangzhou, 510282 China; 2 Medical Apparatus and Equipment Deployment, Zhujiang Hospital, Southern Medical University, Guangzhou, China; 3 Department of Organ Transplantation, Zhujiang Hospital, Southern Medical University, Guangzhou, China; 4 Department of Cardiology, Sun Yat-sen Memorial Hospital, Sun Yat-sen University, Guangzhou, 510120 China

**Keywords:** Cardiovascular diseases, Remnant cholesterol, Risk factors, Aortic valve calcium

## Abstract

**Background:**

Remnant cholesterol (RC) is implicated in the risk of cardiovascular disease. However, comprehensive population-based studies elucidating its association with aortic valve calcium (AVC) progression are limited, rendering its precise role in AVC ambiguous.

**Methods:**

From the Multi-Ethnic Study of Atherosclerosis database, we included 5597 individuals (61.8 ± 10.1 years and 47.5% men) without atherosclerotic cardiovascular disease at baseline for analysis. RC was calculated as total cholesterol minus high-density lipoprotein cholesterol (HDL-C) and low-density lipoprotein cholesterol (LDL-C), as estimated by the Martin/Hopkins equation. Using the adjusted Cox regression analyses, we examined the relationships between RC levels and AVC progression. Furthermore, we conducted discordance analyses to evaluate the relative AVC risk in RC versus LDL-C discordant/concordant groups.

**Results:**

During a median follow-up of 2.4 ± 0.9 years, 568 (10.1%) participants exhibited AVC progression. After adjusting for traditional cardiovascular risk factors, the HRs (95% CIs) for AVC progression comparing the second, third, and fourth quartiles of RC levels with the first quartile were 1.195 (0.925–1.545), 1.322 (1.028–1.701) and 1.546 (1.188–2.012), respectively. Notably, the discordant high RC/low LDL-C group demonstrated a significantly elevated risk of AVC progression compared to the concordant low RC/LDL-C group based on their medians (HR, 1.528 [95% CI 1.201–1.943]). This pattern persisted when clinical LDL-C threshold was set at 100 and 130 mg/dL. The association was consistently observed across various sensitivity analyses.

**Conclusions:**

In atherosclerotic cardiovascular disease-free individuals, elevated RC is identified as a residual risk for AVC progression, independent of traditional cardiovascular risk factors. The causal relationship of RC to AVC and the potential for targeted RC reduction in primary prevention require deeper exploration.

**Supplementary Information:**

The online version contains supplementary material available at 10.1186/s12933-023-02081-2.

## Background

Aortic valve calcium (AVC) is characterized by the pathological calcification of the aortic valve, evolving into stenosis and ultimately manifesting as calcific aortic valve disease [1, 2]. This condition stands as the third leading cardiovascular disorder in the Western world, surpassed only by coronary heart disease and hypertension [3]. Notably, no reliable medical treatment available to prevent the development or progression of AVC. When the disease intensifies to severe aortic stenosis, the sole therapeutic option remains aortic valve replacement, which coupled with potential surgical complications, imposes considerable healthcare financial burdens [4, 5]. There is a pressing imperative to develop efficacious pharmacological measures to thwart the progression of this disease. Consequently, identifying the risk factors for AVC progression potentially elucidates the avenues for innovative preventive and treatment modalities.

Currently, AVC is understood as a complex, multi-step process and exhibits both epidemiologic and histopathologic parallels with atherosclerosis. Risk factors, such as hyperlipidemia, play pivotal roles in AVC development [6, 7]. Hyperlipidemia chiefly impacts the progression of the disease by promoting pro-inflammatory molecules and lipid accumulation, culminating in the irreversible calcification of aortic valve leaflets [8]. Epidemiological studies have pinpointed low-density lipoprotein cholesterol (LDL-C) as a key risk factor for calcific aortic valve disease [9, 10]. However, large-scale randomized trials targeting LDL-C reduction in patients with advanced calcific aortic valve disease have not efficacy in halting the disease's progression [11–13]. This suggests the presence of distinct pathogenetic mechanisms behind AVC that remain to be uncovered. Recent focus has shifted to remnant cholesterol (RC), which has been shown to play a crucial role in the incidence of atherosclerotic cardiovascular disease, and also contributes to aortic valve stenosis [14, 15]. RC is defined as total cholesterol (TC) minus high-density lipoprotein cholesterol (HDL-C) minus LDL-C [16]. Our previous study reveals that high RC levels correlate with an uptick in coronary artery calcium progression, regardless of other traditional cardiovascular risk factors and even among those with ideal LDL-C control [17]. Additionally, observational studies demonstrated a potential link between RC and bioprosthetic valve calcification [18]. While the association between RC and coronary artery disease (CAD), as highlighted in the Multi-Ethnic Study of Atherosclerosis (MESA), is well-established [19], the prospective relationship between RC levels and AVC progression is unclear. Given the shared risk factors between CAD and AVC, particularly in individuals with advanced atherosclerosis [20], the exploration of RC as an independent risk factor for AVC progression, may broaden the current understanding of RC in cardiovascular diseases besides CAD.

In this study, we aimed to investigate the relationship between RC levels and AVC progression within a population-based prospective cohort of Black and White Americans. In addition, we assessed the influence of RC levels on AVC progression among individuals exhibiting optimal LDL-C levels, seeking to determine its potential role in the persistence of calcific aortic valve disease.

## Methods

Data supporting this study’s findings can be obtained from the corresponding author upon a reasonable request.

### Setting

In this study, we utilized individual-level data from the MESA, a landmark US cohort study. Details of the MESA study have been previously published [21]. This approach was adopted to enhance the precision and generalizability of our findings. All study protocols received approval from the institutional review boards at each participating institution. Furthermore, every participant provided written informed consent during their respective study visits.

### Study population

**Fig. 1:**
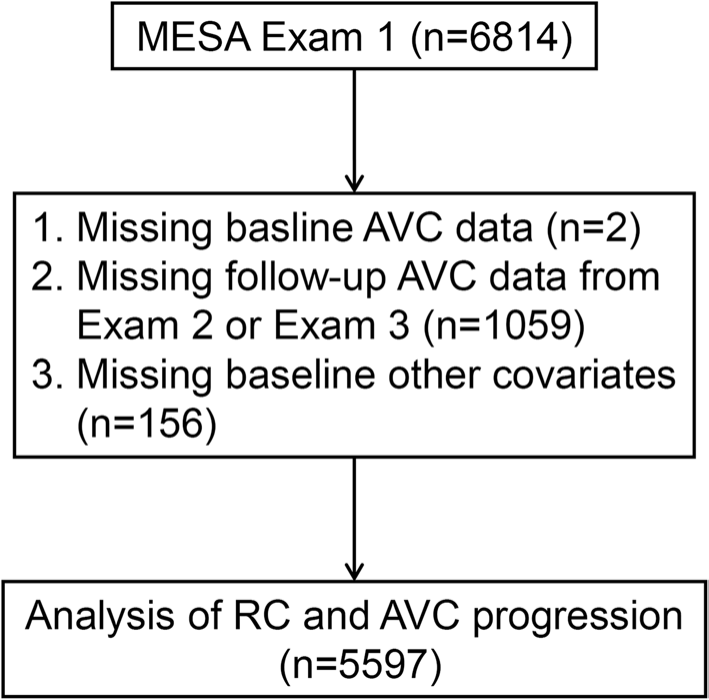
Flowchart for selecting the participants for analysis. *MESA* Multi-Ethnic Study of Atherosclerosis, *AVC* aortic valve calcium, *RC* remnant cholesterol

For the present analysis, the baseline visit is denoted as the initial AVC measurement during 2000–2002, encompassing the MESA Exam 1 cohort (n = 6814). All baseline participants underwent an inclusion screening. As illustrated in Fig. 1, participants were excluded if any of the following data was unavailable: baseline AVC data (n = 2), follow-up AVC data from Exam 2 or Exam 3 (n = 1059), or other pertinent covariates (n = 156). The final study sample for this analysis was 5597 participants.

### Measurements of lipid levels

Fasting blood samples were collected, stored at − 70 °C, and subsequently analyzed in a central laboratory within approximately 2 weeks of collection. TC and HDL-C were quantified using cholesterol oxidase methods, while triglycerides (TG) were assessed with the TG GB reagent. All measurements were conducted on a Roche COBAS FARA centrifugal analyzer (Roche Diagnostics, Indianapolis, IN, USA). Notably, HDL-C quantification followed the precipitation of non–HDL-C by magnesium/dextran (Roche Diagnostics).

Recognizing the limitations of the Friedewald equation, particularly its propensity to underestimate LDL-C in the presence of hypertriglyceridemia or when TG levels are ≥ 150 mg/dL [22], we employed the Martin/Hopkins equation. This equation estimates LDL-C as (non-HDL-C) minus (TG/adjustable factor mg/dL), where the adjustable factor is chosen from one of 180 stratifications according to non-HDL-C and TG levels [23]. These adjustable factor was determined as the strata-specific median TG to very-low-density lipoprotein cholesterol ratio, among these stratifications. Notably, this method has received external validation from groups both within and outside the US [24, 25]. To compute RC levels, we used the equation: RC = TC minus HDL-C minus calculated LDL-C [26]. While no standardized method exists for estimating RC, this approach, derived from the standard lipid profile, has been frequently adopted in a series of prior research [27, 28]. Additionally, it has been validated that utilizing the Martin/Hopkins estimation for LDL-C yields a more accurate RC estimation compared to the Friedewald method [29]. Non-HDL-C was determined by subtracting HDL-C from TC.

### AVC measurements

The methodology used in the MESA study has been detailed previously, with calcified lesions of the aortic valve leaflets and those extending from the aortic valve into the aortic root classified as AVC [30]. The Agatston method was used to quantify AVC. AVC was defined as absent (AVC = 0) or prevalent (AVC > 0). Scans were processed and interpreted at a centralized reading center (Harbor University of California, Los Angeles), and there was low intrareader and interscan variability of 4.4 and 9.7%, respectively, for AVC scoring [31]. The follow-up AVC measurements were conducted at exam 2 (2002–2004), exam 3 (2004–2005). AVC progression was identified by: (1) AVC > 0 at follow-up for participants with a baseline AVC = 0; (2) an annualized change of ≥ 0 at follow-up for those with baseline AVC > 0.

### Measurements of other covariates

We collected comprehensive data on participants' demographic and anthropometric features, lifestyles, and cardiovascular risk factors. These included age, race/ethnicity, sex, and smoking and drinking habits (grouped as never, former, or current). Diabetes was ascertained through fasting glucose (FG) levels ≥ 126 mg/dL, non-FG > 200 mg/dL, prior diagnosis, or use of hypoglycemic medications. Hypertension was indicated by antihypertensive medication use, a hypertension diagnosis, or three consecutive systolic blood pressure (SBP) ≥ 140 mmHg or diastolic blood pressure (DBP) ≥ 90 mmHg. Detailed descriptions of measurements of body mass index (BMI), waist circumference (WC), SBP, DBP, C-reactive protein (CRP), FG, and estimate glomerular filtration rate (eGFR) of the participants have been published previously [32, 33].

### Statistical analysis

Continuous data with normal distributions were presented as mean ± SD, and those without as median (interquartile range). Categorical data were noted as numbers (percentages). Clinical characteristics were analyzed across RC quartiles using χ^2^ tests, ANOVA, or the Kruskal–Wallis *h* test, as fitting. Follow-up ranged from baseline until the earliest of AVC progression, loss to follow-up, or study conclusion. The Cox regression estimated AVC progression risk linked to RC, expressed as HRs and 95% CIs. Multivariate models were adjusted for factors like age, antihypertensive medication use, BMI, CRP, drinking status, eGFR, FG, hypoglycemic medication use, lipid-lowering medication use, race, SBP, sex, and smoking status. A multivariate- adjusted restricted cubic spline regression with 3 knots depicted the dose–response relationship between RC and AVC progression. Sensitivity analyses were conducted as follow: (1) excluded those using lipid-lowering medications; (2) using the Fine-Gray model to account for competitive mortality risk. Moreover, subgroup analyses were segmented by parameters such as traditional risk factors and baseline AVC status.

Due to the absence of physiological cut points for discordance among lipid or lipoprotein measures, we adopted multiple approaches to define discordance. Initially, as mirrored in existing literature [34], we defined discordance using median cut points. Additionally, we applied clinical LDL-C cut points of 100, and 130 mg/dL [35, 36]. Corresponding population percentiles from the cohort determined RC cut points relative to these LDL-C values [26]. Four distinct concordance/discordance categories arose based on RC and LDL-C level cut points: low/low (below-cut points for both), low/high (below for RC, at or above for LDL-C), high/low (at or above for RC, below for LDL-C), and high/high (at or above for both).

Finally, all the analyses were performed using SPSS (version 23, SPSS, Inc, Chicago, IL) and R software (version 3.6.1, R Foundation for Statistical Computing, Vienna, Austria). A *P* value of < 0.05 was deemed statistically significant.

## Results

### Baseline characteristics according to quartiles of RC levels

The baseline characteristics of all the included participants are presented in Table 1. The average age at baseline was 61.8 ± 10.1 years, 2660 (47.5%) were men, 2214 (39.6%) were Caucasian, 667 (11.9%) were Chinese, 1510 (27.0%) were African American, 1206 (21.5%) were Hispanic, 2429 (43.4%) had hypertension and 655 (11.7%) had diabetes. In addition, 698 (12.5%) participants had detectable AVC at baseline, and the mean RC was 23.3 ± 8.4 mg/dL.

**Table 1:** Baseline characteristics of participants stratified by RC quartile groups

Characteristics	Total (n = 5597)	Quartile 1 (n = 1409)	Quartile 2 (n = 1377)	Quartile 3 (n = 1411)	Quartile 4 (n = 1400)	*P* value
RC, mg/dL	23.3 ± 8.4	14.6 ± 1.8	19.2 ± 1.3	24.4 ± 1.7	35.0 ± 6.3	< 0.001
Age, years	61.8 ± 10.1	61.8 ± 10.6	62.2 ± 10.1	62.2 ± 10.1	61.2 ± 9.7	0.034
Men, n (%)	2660 (47.5%)	662 (47.0%)	637 (46.3%)	658 (46.6%)	703 (50.2%)	0.135
Race, n (%)						< 0.001
Caucasian	2214 (39.6%)	554 (39.3%)	515 (37.4%)	567 (40.2%)	578 (41.3%)	
Chinese	667 (11.9%)	125 (8.9%)	153 (11.1%)	185 (13.1%)	204 (14.6%)	
African American	1510 (27.0%)	560 (39.7%)	450 (32.7%)	320 (22.7%)	180 (12.9%)	
Hispanic	1206 (21.5%)	170 (12.1%)	259 (18.8%)	339 (24.0%)	438 (31.3%)	
Waist circumference, cm	97.9 ± 14.4	93.7 ± 14.6	97.2 ± 14.5	99.4 ± 14.2	101.5 ± 12.9	< 0.001
BMI, kg/m^2^	28.3 ± 5.4	27.1 ± 5.4	28.0 ± 5.6	28.7 ± 5.4	29.3 ± 5.0	< 0.001
SBP, mmHg	125.8 ± 21.0	123.3 ± 21.9	125.7 ± 21.4	126.4 ± 20.3	127.7 ± 20.2	< 0.001
DBP, mmHg	71.8 ± 10.1	71.1 ± 10.1	71.7 ± 10.3	71.7 ± 10.1	72.6 ± 9.9	0.002
Smoking status, n (%)						0.034
Never smoker	2829 (50.5%)	690 (49.0%)	706 (51.3%)	716 (50.7%)	717 (51.2%)	
Former smoker	2080 (37.2%)	571 (40.5%)	502 (36.5%)	517 (36.6%)	490 (35.0%)	
Current smoker	688 (12.3%)	148 (10.5%)	169 (12.3%)	178 (12.6%)	193 (13.8%)	
Drinking status, n (%)						< 0.001
Never drinker	1128 (20.2%)	228 (16.2%)	260 (18.9%)	320 (22.7%)	320 (22.9%)	
Former drinker	1289 (23.0%)	340 (24.1%)	322 (23.4%)	323 (22.9%)	304 (21.7%)	
Current drinker	3180 (56.8%)	841 (59.7%)	795 (57.7%)	768 (54.4%)	776 (55.4%)	
Hypertension, n (%)	2429 (43.4%)	534 (37.9%)	609 (44.2%)	641 (45.4%)	645 (46.1%)	< 0.001
Diabetes, n (%)	655 (11.7%)	122 (8.7%)	131 (9.5%)	172 (12.2%)	230 (16.4%)	< 0.001
Antihypertensive, n (%)	2018 (36.1%)	466 (33.1%)	517 (37.5%)	521 (36.9%)	514 (36.7%)	0.058
Hypoglycemic medication, n (%)	499 (8.9%)	97(6.9%)	102 (7.4%)	126 (8.9%)	174 (12.4%)	< 0.001
Lipid-lowering medication, n (%)	910 (16.3%)	205 (14.5%)	228 (16.6%)	221 (15.7%)	256 (18.3%)	0.052
CRP, mg/L	1.9 (0.8, 4.2)	1.3 (0.6, 3.3)	1.8 (0.8, 4.1)	2.0 (0.9, 4.5)	2.4 (1.1, 4.7)	< 0.001
TG, mg/dL	125.7 ± 65.5	60.2 ± 12.5	93.5 ± 11.1	132.5 ± 17.8	216.7 ± 54.0	< 0.001
TC, mg/dL	193.6 ± 34.1	178.5 ± 29.7	188.4 ± 31.2	196.6 ± 32.1	210.8 ± 34.7	< 0.001
HDL-C, mg/dL	51.1 ± 14.6	59.8 ± 15.8	53.0 ± 13.6	48.5 ± 12.7	43.2 ± 10.3	< 0.001
LDL-C, mg/dL	119.1 ± 30.3	104.1 ± 26.4	116.2 ± 27.7	123.6 ± 28.5	132.6 ± 30.8	< 0.001
Non-HDL-C, mg/dL	142.4 ± 34.2	118.7 ± 27.0	135.4 ± 27.8	148.0 ± 28.7	167.6 ± 32.7	< 0.001
FG, mg/dL	96.3 ± 27.8	91.3 ± 22.1	94.0 ± 22.5	97.4 ± 28.8	102.5 ± 34.7	< 0.001
eGFR, ml/min/1.73 m^2^	78.1 ± 16.0	79.1 ± 15.8	78.4 ± 15.9	77.5 ± 15.9	77.4 ± 16.4	0.042
Baseline AVC, n (%)	698 (12.5%)	145 (10.3%)	157 (11.4%)	196 (13.9%)	200 (14.3%)	0.002

*AVC* aortic valve calcium, *BMI* body mass index, *CRP* C-reactive protein, *DBP* diastolic blood pressure, *eGFR* estimate glomerular filtration rate, *FG* fasting glucose, *HDL-C* high-density lipoprotein cholesterol, *LDL-C* low-density lipoprotein cholesterol, *RC* remnant cholesterol, *SBP* systolic blood pressure, *TC* total cholesterol, *TG* triglycerides, *WC* waist circumference

The individuals were categorized into 4 groups based on the quartiles of baseline RC levels (Table 1). Participants with a higher RC level were more likely to be Caucasian and to have higher levels of waist circumference, BMI, SBP, DBP, CRP, TG, TC, LDL-C, non-HDL-C, and FG but had lower levels of HDL-C and eGFR. Likewise, participants in a higher RC quartile had a higher prevalence of hypertension, diabetes, and AVC and were more likely to take hypoglycemic medication. The comparison of baseline characteristics between participants included and excluded in the analysis are reported in Additional file 1: Table S1. Excluded participants were more often female, African American, current smoker, former drinker, and had AVC at baseline. They were also more likely to take antihypertensive and hypoglycemic medication and had higher levels of age, BMI, SBP, CRP, TG, HDL-C, and FG.

### Association between RC levels and AVC progression

**Fig. 2:**
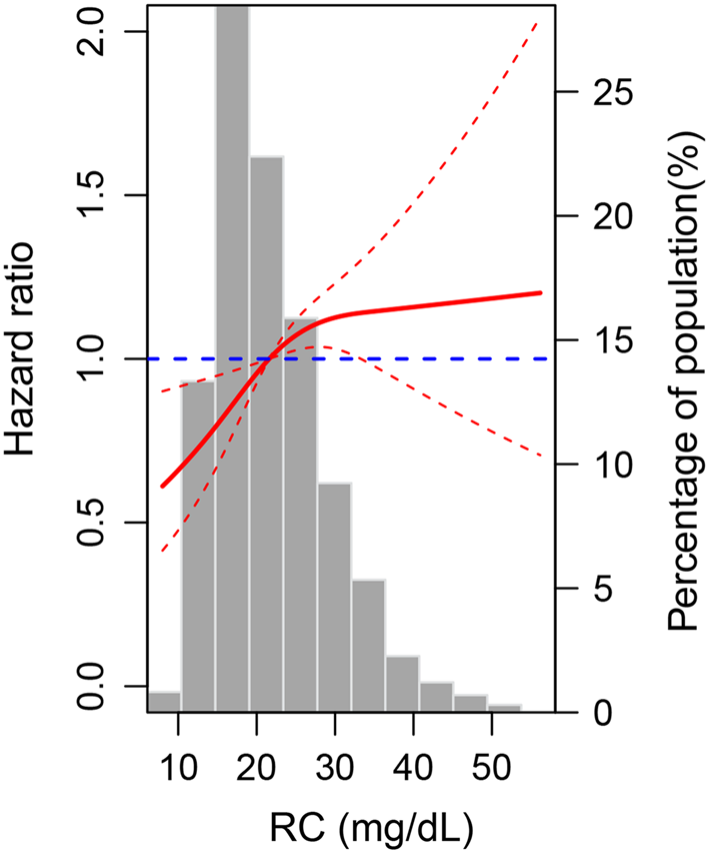
Adjusted hazard ratios of aortic valve calcium progression by remnant cholesterol (RC). Each hazard ratio was computed with an RC level of 21.6 mg/dL as the reference. The hazard ratio was adjusted for age, antihypertensive medication use, body mass index, C-reactive protein, drinking status, estimate glomerular filtration rate, fasting glucose, hypoglycemic medication use, lipid-lowering medication use, race, sex, smoking status, and systolic blood pressure. The red solid line represents the hazard ratio of RC across the whole range. The red dotted line represented the 95% confidence interval. The blue dotted line was the reference line with a hazard ratio of 1. Histograms represented the frequency distribution of RC

During a follow-up period of 2.4 ± 0.9 years, 568 (10.1%) cases exhibited AVC progression. The percentage of AVC progression increased with increasing quartiles of RC levels (quartiles 1–4: 110 [7.8%] versus 135 [9.8%] versus 160 [11.3%] versus 151 [11.6%], *P* = 0.003, Table 2). In the fully adjusted model, the HRs (95% CIs) for AVC progression comparing the second, third, and fourth quartiles of RC levels with the first quartile were 1.195 (0.925–1.545), 1.322 (1.028–1.701) and 1.546 (1.188–2.012), respectively (Table 2). The sensitivity analysis showed that the significant associations of RC and AVC progression remained after further adjustment for LDL-C, and HDL-C levels separately (models 3–4 in Table 2). Multivariable-adjusted restricted cubic splines regression models analysis that RC levels were positively and nonlinearly associated with AVC progression, compared with the reference of 21.6 mg/dL (Fig. 2).

**Table 2:** Risk of AVC progression for RC quartile groups

RC	Events/No. at risk	Model 1 HR (95% CI)	*P* value	Model 2 HR (95% CI)	*P* value	Model 3 HR (95% CI)	*P* value	Model 4 HR (95% CI)	*P* value
Quartile 1	110/1409	Reference	1.0	Reference	1.0	Reference	1.0	Reference	1.0
Quartile 2	135/1377	1.251 (0.970–1.614)	0.085	1.195 (0.925–1.545)	0.173	1.174 (0.906–1.522)	0.225	1.169 (0.899–1.520)	0.245
Quartile 3	160/1411	1.428 (1.116–1.829)	0.005	1.322 (1.028–1.701)	0.030	1.282 (0.988–1.664)	0.062	1.277 (0.977–1.667)	0.073
Quartile 4	163/1400	1.755 (1.363–2.261)	< 0.001	1.546 (1.188–2.012)	0.001	1.481 (1.120–1.958)	0.006	1.474 (1.104–1.968)	0.008
RC, mg/dL	568/5597	1.021 (1.011–1.031)	< 0.001	1.016 (1.005–1.026)	0.004	1.013 (1.002–1.025)	0.022	1.013 (1.002–1.025)	0.025

Model 1: Adjusted for age, race and sex. Model 2: Adjusted for Model 1 covariates plus antihypertensive medication use, BMI, CRP, drinking status, eGFR, FG, hypoglycemic medication use, lipid-lowering medication use, SBP and smoking status. Model 3: Adjusted for Model 2 covariates plus LDL-C. Model 4: Adjusted for Model 2 covariates plus HDL-C

*AVC* aortic valve calcium, *BMI* body mass index, *CI* confidence interval, *CRP* C-reactive protein, *eGFR* estimate glomerular filtration rate, *FG* fasting glucose, *HDL-C* high-density lipoprotein cholesterol, *HR* hazard ratio, *LDL-C* low-density lipoprotein cholesterol, *RC* remnant cholesterol, *SBP* systolic blood pressure

In the subsample of participants without lipid-lowering therapy at baseline (n = 4687), there was still a significant association between RC and the risk of AVC progression in the full adjustments (HR [95% CI] for quartile 2: 1.453 [1.070–1.972]; quartile 3: 1.556 [1.148–2.108]; and quartile 4: 1.758 [1.278–2.417]; Additional file 1: Table S2). Similar results were found after further adjustment for lipid-lowering medication use during follow-up (HR [95% CI] for quartile 2: 1.577 [1.141–2.180]; quartile 3: 1.683 [1.224–2.313]; and quartile 4: 1.776 [1.267–2.489]; n = 4241; Additional file 1: Table S2) or in those free of lipid-lowering medication use throughout (HR [95% CI] for quartile 2: 1.537 [1.055–2.239]; quartile 3: 1.633 [1.123–2.376] and quartile 4: 1.951 [1.340–2.842]; n = 3435; Additional file 1: Table S2). Using the Fine and Gray method to assess the relationship between RC levels and AVC progression also produced similar results (Additional file 1: Fig S1).

**Fig. 3:**
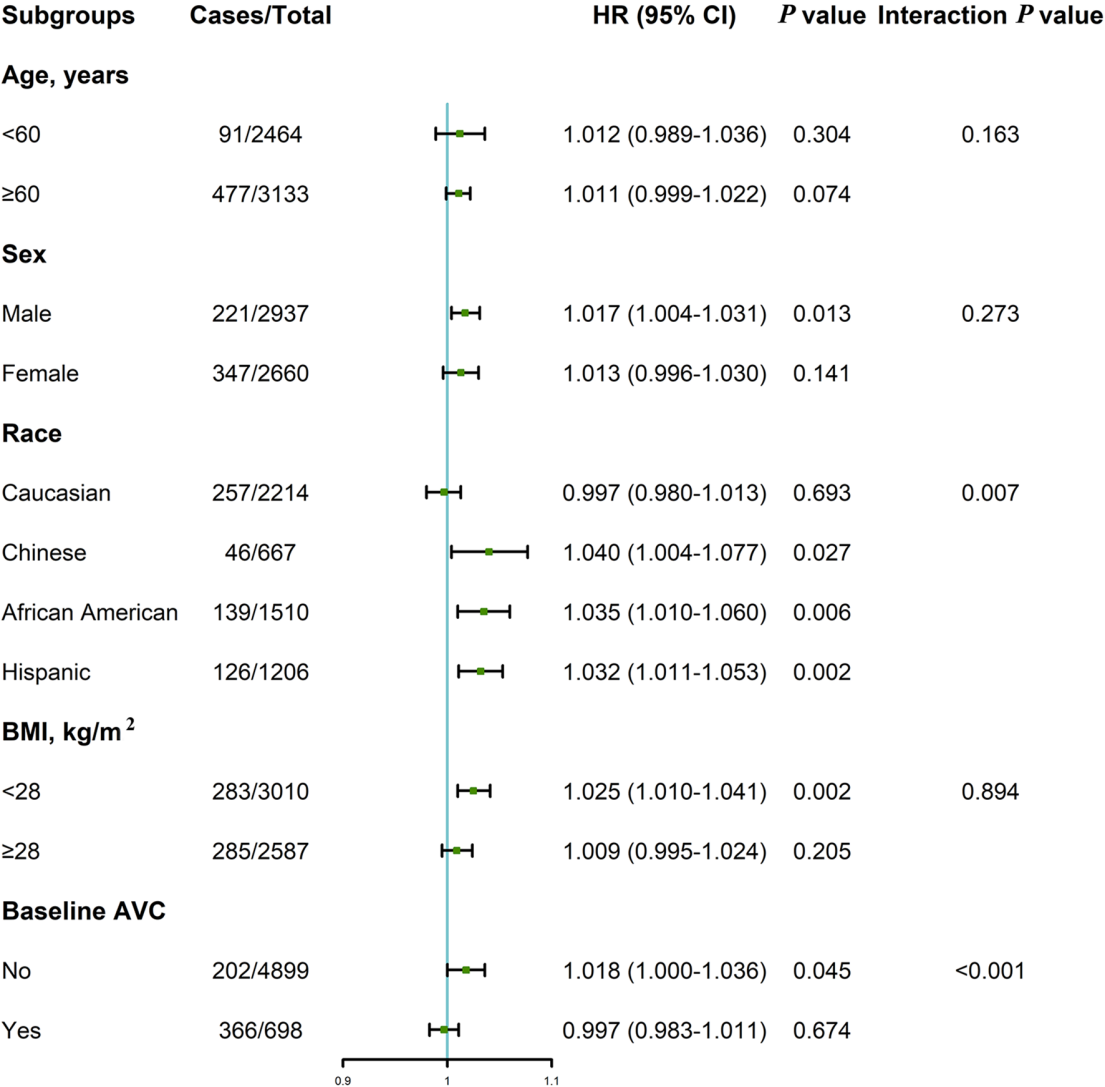
Subgroup analysis of the association between remnant cholesterol and aortic valve calcium (AVC) progression. Cox regression was performed after adjustment for age, antihypertensive medication use, body mass index, C-reactive protein, drinking status, estimate glomerular filtration rate, fasting glucose, hypoglycemic medication use, lipid-lowering medication use, race, sex, smoking status, and systolic blood pressure. *HR* hazard ratio, *BMI* body mass index, *CI* confidence interval

When participants were stratified by age (< 60 or ≥ 60 years), sex (male or female), and BMI (< 28 or ≥ 28 kg/m^2^), the association between RC levels and AVC progression remained similar among these subgroups (Fig. 3). However, a differential association was observed if subgroups were divided by race (Caucasian, Chinese, African American or Hispanic) and baseline AVC status (yes or no), showing a stronger positive association between RC levels and AVC progression in Chinese, African American and Hispanic than in Caucasian (*P* for interaction = 0.007, Fig. 3). The positive association between RC levels and AVC progression was also stronger in participants without AVC than in participants with AVC at baseline (*P* for interaction < 0.001, Fig. 3).

### Comparison of the roles of RC in AVC progression with other lipid fractions

In a comparison of individuals in the top 3 quartiles with those in the bottom quartile of TG levels, the multivariable-adjusted HRs (95% CIs) for AVC progression were 1.064 (0.823–1.377), 1.226 (0.952–1.578), and 1.409 (1.085–1.830) in the second, third, and fourth quartiles, respectively (Additional file 1: Table S4). Similar overall findings were observed for the association between elevated non-HDL-C levels and the risk of AVC progression (Additional file 1: Table S5). However, there was no significant association between elevated LDL-C levels and the risk of AVC progression (Additional file 1: Table S3).

In the discordance analysis defined by median cut points, we demonstrated a significantly higher risk of AVC progression in the discordant high RC/low LDL-C group than in the concordant low RC/LDL-C group (HR, 1.528 [95% CI 1.201–1.943]) after adjustment for traditional cardiovascular risk factors (Table 3). We observed similar findings when excluding individuals on lipid-lowering therapy at baseline (Additional file 1: Table S6). Likewise, the discordant high RC/low LDL-C group still exhibited a significantly higher risk of AVC progression than the concordant low RC/LDL-C group in the fully adjusted model regarding LDL-C cut points of 100 mg/dL (HR, 1.604 [95% CI 1.006–2.558]) and 130 mg/dL (HR, 1.589 [95% CI 1.210–2.088]; Additional file 1: Table S7).

**Table 3:** Risk of AVC progression across LDL-C vs RC concordant/discordant groups by LDL-C median and RC median

LDL-C groups	RC groups	Events/No. at risk	Model 1 HR (95% CI)	*P* value	Model 2 HR (95% CI)	*P* value	Model 3 HR (95% CI)	*P* value
LDL-C, mg/dL								
< 117.6	–	290/2794	Reference	1.0	Reference	1.0	Reference	1.0
≥ 117.6	–	278/2803	0.972 (0.824–1.147)	0.736	1.060 (0.894–1.257)	0.500	1.057 (0.891–1.254)	0.525
RC, mg/dL								
–	< 21.6	245/2786	Reference	1.0	Reference	1.0	Reference	1.0
–	≥ 21.6	323/2811	1.389 (1.171–1.647)	< 0.001	1.284 (1.077–1.531)	0.005	1.280 (1.073–1.527)	0.006
Cut points: LDL-C, 117.6 mg/dL; RC, 21.6 mg/dL								
< Cut point	< Cut point	150/1743	Reference	1.0	Reference	1.0	Reference	1.0
	≥ Cut point	140/1051	1.718 (1.359–2.171)	< 0.001	1.529 (1.203–1.945)	0.001	1.528 (1.201–1.943)	0.001
≥ Cut point	< Cut point	95/1043	1.108 (0.856–1.434)	0.434	1.230 (0.948–1.598)	0.119	1.231 (0.948–1.598)	0.119
	≥ Cut point	183/1760	1.287 (1.034–1.603)	0.024	1.295 (1.034–1.621)	0.024	1.289 (1.029–1.616)	0.027

Model 1: Adjusted for age, race and sex. Model 2: Adjusted for Model 1 covariates plus antihypertensive medication use, BMI, drinking status, FG, hypoglycemic medication use, lipid-lowering medication use, SBP and smoking status. Model 3: Adjusted for Model 2 covariates plus CRP and eGFR

*AVC* aortic valve calcium, *BMI* body mass index, *CI* confidence interval, *CRP* C-reactive protein, *eGFR* estimate glomerular filtration rate, *FG* fasting glucose, *HDL-C* high-density lipoprotein cholesterol, *HR* hazard ratio, *LDL-C* low-density lipoprotein cholesterol, *RC* remnant cholesterol, *SBP* systolic blood pressure

## Discussion

In a diverse sample from US community-based cohorts with a median follow-up of 2.4 ± 0.9 years, we found that (1) elevated RC levels significantly correlate with AVC progression, independent of traditional cardiovascular factors; (2) this association is more pronounced in Chinese, African American, and Hispanic populations, and those without AVC at baseline; (3) the relationship remains significant even at optimal LDL-C levels; (4) individuals with high RC/low LDL-C discordance have a heightened AVC risk compared to those with harmonized levels. These findings highlight the importance of addressing RC risk in the era of targeted lipid-lowering therapies. Future studies should elucidate the mechanisms behind RC’s role in AVC and determine if reducing RC levels improves outcomes.

Epidemiological studies have emphasized LDL-C's role in AVC risk [37, 38]. While observational data hinted at a decreased occurrence and progression of aortic stenosis in statin users [39], subsequent large-scale randomized trials did not support this [40, 41]. Such inconsistencies challenged the LDL hypothesis in aortic valve disease and temper the enthusiasm for LDL-C reduction as a preventive strategy. Emerging evidence indicates that RC is a key player in the residual risk for arteriosclerotic cardiovascular disease. This connection gains significance against the backdrop of the rising global prevalence of obesity, diabetes, and metabolic syndrome—all linked to elevated RC levels and its potential overlap with calcific aortic valve disease. In this context, our study offers the first population-based evidence that each 1 mg/dL increase in RC corresponds to a 1.6% heightened risk of AVC progression, a notable predictor of calcific aortic valve disease.

In understanding the intricate dynamics of AVC progression, cholesterol and TG, both nonpolar and water-insoluble lipids, necessitate lipoprotein particles for plasma transport [42]. RC primarily encompasses plasma cholesterol not encapsulated within LDL-C or HDL-C bounds and predominantly consists of triglyceride-rich lipoproteins (TGRL). Elevated TG often serves as an indicator of increased RC, making plasma TG a symbolic measure for both TGRL and RC. Some argue that RC could just be an alternative term for TG in LDL-C computation, emphasizing the intrinsic biological connection between RC and TG [43]. However, a recent one-sample Mendelian randomization study involving 473 aortic stenosis cases underscored the causal relationship between LDL-C and aortic stenosis [44]. The nonexistence of a notable correlation with TG may stem from the constrained ability to discern minor effect sizes, an inherent shortcoming of one-sample Mendelian randomization [45]. TG was directly involved in calcific aortic valve disease compared to LDL-C, but they may represent RC levels or, synonymously, the abundance of TGRL particles. The critical role of cholesterol in TGRL concerning calcific aortic valve disease is pivotal, given its accumulation in the intima, unlike TG.

Our study underscored that the group with discordant high RC levels but low LDL-C exhibited an amplified risk for AVC progression relative to the concordant group, as determined by both median and clinical LDL-C benchmarks. Importantly, RC displayed a more pronounced detrimental effect on AVC progression than LDL-C, pointing to distinct underlying mechanisms for RC-induced AVC exacerbation. RC seamlessly enters the arterial wall, being absorbed by macrophages and smooth muscle cells without any prior modification, a behavior not mirrored by LDL-C, which demands pre-uptake modification [46]. Furthermore, remnant particles, possessing up to 40 times the cholesterol per particle and being larger than LDL, might have a superior atherogenic capacity than LDL-C [47]. Elevated RC levels, unlike LDL-C levels, were correlated with the low-grade inflammation seen in ischemic heart disease [42]. For those at low-to-moderate risk, an LDL-C target of < 100 mg/dL is advocated in primary prevention. We postulate that certain participants might be prescribed advanced lipid-lowering agents, such as proprotein convertase subtilisin/kexin type 9 inhibitors, targeting even stricter LDL-C levels (e.g., < 70 mg/dL), thereby concurrently reducing RC levels [35]. Interestingly, the association between RC and AVC progression was notably more pronounced in participants who did not have AVC at baseline. Additionally, this association seemed to be stronger among Chinese, African American, and Hispanic participants, suggesting that these groups may be more susceptible to the influence of RC on AVC progression [48]. Such findings corroborate the prevailing view that a well-balanced lipid profile is associated with long-term non-development of AVC. While statins may hasten plaque calcification [49], their concurrent plaque-stabilizing and regressing capabilities can introduce ambiguity in assessing AVC severity. However, our subsequent analyses confirmed the robustness of the RC-AVC progression relationship, even among those without statins use.

In the emerging era of targeted RC-lowering therapies, understanding RC’s residual risk is crucial. For RC’s seamless clinical integration, a consensus on its efficient and cost-effective measurement is vital. Future studies must clarify how RC relates to calcific aortic valve disease, and determine if targeted RC reductions yields cardiovascular benefits.

### Study limitations

By leveraging data from representative landmark US cohorts, we achieved enhanced generalizability over other epidemiological studies. We not only emphasized RC’s independent predictive value as a continuous measure but also elucidated its augmented risk using a discordance analysis across multiple clinical LDL-C thresholds, reinforcing the rigor of our results. Crucially, the Martin/Hopkins equation used for LDL-C estimation yielded more accurate RC values than the Friedewald equation. However, several limitations should be highlighted, the potential residual confounding remained despite of multiple adjustment because of the nature of observational design. While our extended follow-up offers robust insights, significant lipid level fluctuations over time are undeniable. Due to data constraints at baseline AVC measurements, we were unable to assess the association between TGRL fractions, such as apolipoprotein B, and AVC progression. Additionally, the relationship between lipoprotein(a) and RC in the AVC milieu remains an intriguing topic [50]. Delving deeper into whether lipoprotein(a) works in tandem with RC, operates autonomously, or if they jointly influence overlapping pathways in AVC remains a pertinent avenue for future research.

## Conclusions

In conclusion, in individuals free of atherosclerotic cardiovascular disease, elevated RC is associated with an increased risk of AVC progression irrespective of traditional cardiovascular risk factors, even among those with optimal LDL-C levels.

### Supplementary Information

**Additional file 1: Table S1.** Baseline Characteristics of Included and Excluded Participants. **Table S2.** Risk of AVC Progression for RC Quartile Groups Excluding Those on Lipid-Lowering Therapy at Baseline (model 1, n = 4687 and model 2, n = 4241) and During Follow-Up (model 3, n = 3435). **Table S3.** Risk of AVC Progression for LDL-C Quartile Groups. **Table S4.** Risk of AVC Progression for Triglycerides Quartile Groups. **Table S5.** Risk of AVC Progression for Non-HDL-C Quartile Groups. **Table S6.** Risk of AVC Progression Across LDL-C Versus RC Concordant/Discordant Groups by LDL-C Median and Percentile Equivalents for RC Excluding Those on Lipid-Lowering Therapy at Baseline (Model 1 and Model 2, n = 4687; Model 3, n = 4240). **Table S7.** Risk of AVC Progression Across LDL-C Versus RC Concordant/Discordant groups by LDL-C Clinical Cut Points (100 and 130 mg/dL) and Percentile Equivalents for RC Excluding Those on Lipid-Lowering Therapy at Baseline (Model 1 and Model 2, n = 4210; Model 3, n = 3746). **Figure S1.** Competing risk regression analysis. Cumulative incidence function of follow-up years to AVC progression with all-cause death as a competing risk among different RC quartiles by using the Fine and Gray model. AVC, aortic valve calcium; RC, remnant cholesterol.

## Data Availability

The data that support the findings of this study are available from the corresponding author upon reasonable request. Data described in the manuscript, code book, and analytic code will be available upon request pending application and approval. However, we will make the data used in the manuscript, code book, and analytic code available to editors upon request either before or after publication for checking.

## Abbreviations

AVC: Aortic valve calcium
BMI: Body mass index
CRP: C-reactive protein
DBP: Diastolic blood pressure
eGFR: Estimate glomerular filtration rate
FG: Fasting glucose
HDL-C: High-density lipoprotein cholesterol
HR: Hazard ratio
LDL-C: Low-density lipoprotein cholesterol(a): Lipoprotein(a)
MESA: Multi-ethnic study of atherosclerosis
RC: Remnant cholesterol
SBP: Systolic blood pressure
TC: Total cholesterol
TGRL: Triglyceride-rich lipoprotein
WC: Waist circumference
