# Supplementary material for: Remnant cholesterol and the risk of aortic valve calcium progression: insights from the MESA study
Source: Cardiovasc Diabetol. 2024 Jan 9;23:20. doi: 10.1186/s12933-023-02081-2 (PMC10777602; doi:10.1186/s12933-023-02081-2)
Supplement: Supplementary file 1 — Additional file 1: Table S1. Baseline Characteristics of Included and Excluded Participants. Table S2. Risk of AVC Progression for RC Quartile Groups Excluding Those on Lipid-Lowering Therapy at Baseline (model 1, n = 4687 and model 2, n = 4241) and During Follow-Up (model 3, n = 3435). Table S3. Risk of AVC Progression for LDL-C Quartile Groups. Table S4. Risk of AVC Progression for Triglycerides Quartile Groups. Table S5. Risk of AVC Progression for Non-HDL-C Quartile Groups. Table S6. Risk of AVC Progression Across LDL-C Versus RC Concordant/Discordant Groups by LDL-C Median and Percentile Equivalents for RC Excluding Those on Lipid-Lowering Therapy at Baseline (Model 1 and Model 2, n = 4687; Model 3, n = 4240). Table S7. Risk of AVC Progression Across LDL-C Versus RC Concordant/Discordant groups by LDL-C Clinical Cut Points (100 and 130 mg/dL) and Percentile Equivalents for RC Excluding Those on Lipid-Lowering Therapy at Baseline (Model 1 and Model 2, n = 4210; Model 3, n = 3746). Figure S1. Competing risk regression analysis. Cumulative incidence function of follow-up years to AVC progression with all-cause death as a competing risk among different RC quartiles by using the Fine and Gray model. AVC, aortic valve calcium; RC, remnant cholesterol. [file 12933_2023_2081_MOESM1_ESM.docx]

Supplementary Material

**Table S1. Baseline Characteristics of Included and Excluded Participants**

| **Characteristics** | **Included (n=5597)** | **Excluded (n=1217)** |
| --- | --- | --- |
| Age, years | 61.8±10.1 | 63.6±10.5 |
| Male, n (%) | 2660 (47.5%) | 553 (45.4%) |
| Race, n (%) |  |  |
| Caucasian | 2214 (39.6%) | 408 (33.5%) |
| Chinese | 667 (11.9%) | 137 (11.3%) |
| African American | 1510 (27.0%) | 382 (31.4%) |
| Hispanic | 1206 (21.5%) | 290 (23.8%) |
| BMI, kg/m^2^ | 28.3±5.4 | 28.6±5.6 |
| SBP, mmgHg | 125.8±21.0 | 130.4±23.1 |
| DBP, mmHg | 71.8±10.1 | 72.5±10.8 |
| Smoking status, n (%) |  |  |
| Never smoker | 2829 (50.5%) | 589 (49.3%) |
| Former smoker | 2080 (37.2%) | 407 (34.1%) |
| Current smoker | 688 (12.3%) | 199 (16.7%) |
| Drinking status, n (%) |  |  |
| Never drinker | 1128 (20.2%) | 262 (22.5%) |
| Former drinker | 1289 (23.0%) | 335 (28.7%) |
| Current drinker | 3180 (56.8%) | 569 (48.8%) |
| Hypertension, n (%) | 2429 (43.4%) | 629 (51.7%) |
| Diabetes, n (%) | 655 (11.7%) | 204 (17.1%) |
| Antihypertensive, n (%) | 2018 (36.1%) | 518 (42.7%) |
| Hypoglycemic medication, n (%) | 499 (8.9%) | 156 (12.9%) |
| Lipid-lowering medication, n (%) | 910 (16.3%) | 190 (15.7%) |
| CRP, mg/L | 1.9 (0.8, 4.2) | 2.2 (0.9,4.7) |
| Triglyceride, mg/dL | 125.7±65.5 | 126.5±65.1 |
| TC, mg/dL | 193.6±34.1 | 193.5±37.7 |
| HDL-C, mg/dL | 51.1±14.6 | 51.2±15.5 |
| LDL-C, mg/dL | 119.1±30.3 | 118.8±33.4 |
| FG, mg/dL | 96.3±27.8 | 101.3±37.5 |
| eGFR, ml/min/1.73m^2^ | 78.1±16.0 | 77.7±17.8 |
| Baseline AVC, n (%) | 698 (12.5%) | 215 (17.7%) |

AVC, aortic valve calcium; BMI, body mass index; CRP, C-reactive protein; DBP, diastolic blood pressure; eGFR, estimate glomerular filtration rate; FG, fasting glucose; HDL-C, high-density lipoprotein cholesterol; LDL-C, low-density lipoprotein cholesterol; SBP, systolic blood pressure; TC, total cholesterol.

**Table S2. Risk of AVC Progression for RC Quartile Groups Excluding Those on Lipid-Lowering Therapy at Baseline (model 1, n=4687 and model 2, n=4241) and During Follow-Up (model 3, n=3435)**

| **RC** | **Model 1 HR (95% CI)** | ***P* value** | **Model 2 HR (95% CI)** | ***P* value** | **Model 3 HR (95% CI)** | ***P* value** |
| --- | --- | --- | --- | --- | --- | --- |
| Quartile 1 | Reference | 1.0 | Reference | 1.0 | Reference | 1.0 |
| Quartile 2 | 1.453 (1.070-1.972) | 0.017 | 1.577 (1.141-2.180) | 0.006 | 1.537 (1.055-2.239) | 0.025 |
| Quartile 3 | 1.556 (1.148-2.108) | 0.004 | 1.683 (1.224-2.313) | 0.001 | 1.633 (1.123-2.376) | 0.010 |
| Quartile 4 | 1.758 (1.278-2.417) | 0.001 | 1.776 (1.267-2.489) | 0.001 | 1.951 (1.340-2.842) | <0.001 |

Model 1: Adjusted for age, antihypertensive, BMI, CRP, drinking status, eGFR, FG, hypoglycemic medication use, race, SBP, sex and smoking status. Model 2: Adjusted for model 1 covariates plus lipid-lowering medication use during follow-up. Model 3: Adjusted for model 1 covariates in those free of lipid-lowering medication use throughout. AVC, aortic valve calcium; BMI, body mass index; CI, confidence interval; CRP, C-reactive protein; eGFR, estimate glomerular filtration rate; FG, fasting glucose; HR, hazard ratio; RC, remnant cholesterol; SBP, systolic blood pressure.

**Table S3. Risk of AVC Progression for LDL-C Quartile Groups**

| **RC** | **Model 1 HR (95% CI)** | ***P* value** | **Model 2 HR (95% CI)** | ***P* value** | **Model 3 HR (95% CI)** | ***P* value** |
| --- | --- | --- | --- | --- | --- | --- |
| Quartile 1 | Reference | 1.0 | Reference | 1.0 | Reference | 1.0 |
| Quartile 2 | 0.922 (0.732-1.162) | 0.491 | 0.973 (0.771-1.229) | 0.819 | 0.973 (0.771-1.229) | 0.821 |
| Quartile 3 | 0.832 (0.656-1.055) | 0.129 | 0.931 (0.729-1.188) | 0.564 | 0.930 (0.729-1.188) | 0.563 |
| Quartile 4 | 1.045 (0.829-1.317) | 0.709 | 1.173 (0.925-1.487) | 0.187 | 1.168 (0.920-1.482) | 0.203 |

Model 1: Adjusted for age, race and sex. Model 2: Adjusted for model 1 covariates plus antihypertensive, BMI, drinking status, FG, hypoglycemic medication use, lipid-lowering medication use, SBP and smoking status. Model 3: Adjusted for model 2 covariates plus CRP and eGFR. AVC, aortic valve calcium; BMI, body mass index; CI, confidence interval; CRP, C-reactive protein; eGFR, estimate glomerular filtration rate; FG, fasting glucose; HR, hazard ratio; LDL-C, low-density lipoprotein cholesterol; SBP, systolic blood pressure.

| **Triglycerides** | **Model 1 HR (95% CI)** | ***P* value** | **Model 2 HR (95% CI)** | ***P* value** | **Model 3 HR (95% CI)** | ***P* value** |
| --- | --- | --- | --- | --- | --- | --- |
| Quartile 1 | Reference | 1.0 | Reference | 1.0 | Reference | 1.0 |
| Quartile 2 | 1.133 (0.878-1.462) | 0.339 | 1.068 (0.826-1.381) | 0.615 | 1.064 (0.823-1.377) | 0.635 |
| Quartile 3 | 1.350 (1.054-1.730) | 0.018 | 1.231 (0.95-1.584) | 0.105 | 1.226 (0.952-1.578) | 0.114 |
| Quartile 4 | 1.654 (1.287-2.126) | <0.001 | 1.415 (1.091-1.836) | 0.009 | 1.409 (1.085-1.830) | 0.010 |

**Table S4. Risk of AVC Progression for Triglycerides Quartile Groups**

Model 1: Adjusted for age, race and sex. Model 2: Adjusted for model 1 covariates plus antihypertensive, BMI, drinking status, FG, fasting plasma glucose; hypoglycemic medication use, lipid-lowering medication use, SBP and smoking status. Model 3: Adjusted for model 2 covariates plus CRP and eGFR. AVC, aortic valve calcium; BMI, body mass index; CI, confidence interval; CRP, C-reactive protein; eGFR, estimate glomerular filtration rate; FG, fasting glucose; HR, hazard ratio; SBP, systolic blood pressure.

**Table S5. Risk of AVC Progression for Non-HDL-C Quartile Groups**

| **Non-HDL-C** | **Model 1 HR (95% CI)** | ***P* value** | **Model 2 HR (95% CI)** | ***P* value** | **Model 3 HR (95% CI)** | ***P* value** |
| --- | --- | --- | --- | --- | --- | --- |
| Quartile 1 | Reference | 1.0 | Reference | 1.0 | Reference | 1.0 |
| Quartile 2 | 1.063 (0.838-1.349) | 0.613 | 1.121 (0.882-1.426) | 0.350 | 1.123 (0.882-1.428) | 0.346 |
| Quartile 3 | 0.969 (0.761-1.232) | 0.796 | 1.038 (0.811-1.328) | 0.767 | 1.036 (0.810-1.326) | 0.777 |
| Quartile 4 | 1.301 (1.028-1.647) | 0.028 | 1.430 (1.123-1.821) | 0.004 | 1.425 (1.118-1.816) | 0.004 |

Model 1: Adjusted for age, race and sex. Model 2: Adjusted for model 1 covariates plus antihypertensive, BMI, drinking status, FG, hypoglycemic medication use, lipid-lowering medication use, SBP and smoking status. Model 3: Adjusted for model 2 covariates plus CRP and eGFR. AVC, aortic valve calcium; BMI, body mass index; CI, confidence interval; CRP, C-reactive protein; eGFR, estimate glomerular filtration rate; FG, fasting glucose; HDL-C, high-density lipoprotein cholesterol; HR, hazard ratio; SBP, systolic blood pressure.

**Table S6. Risk of AVC Progression Across LDL-C Versus RC Concordant/Discordant Groups by LDL-C Median and Percentile Equivalents for RC Excluding Those on Lipid-Lowering Therapy at Baseline (Model 1 and Model 2, n=4687; Model 3, n=4240)**

| **LDL-C groups** | **RC groups** | **Events/No. at risk** | **Model 1**  **HR (95% CI)** | ***P* value** | **Model 2**  **HR (95% CI)** | ***P* value** | **Model 3**  **HR (95% CI)** | ***P* value** |
| --- | --- | --- | --- | --- | --- | --- | --- | --- |
| LDL-C, mg/dL | | | | | | | | |
| <120.0 or 119.9 | - | 206/2340 | Reference | 1.0 | Reference | 1.0 | Reference | 1.0 |
| ≥120.0 or 119.9 | - | 213/2347 | 1.103 (0.910-1.338) | 0.316 | 1.116 (0.918-1.357) | 0.269 | 1.076 (0.870-1.331) | 0.501 |
| RC, mg/dL^*^ | | | | | | | | |
| - | <21.5 or 21.4 | 179/2350 | Reference | 1.0 | Reference | 1.0 | Reference | 1.0 |
| - | ≥21.5 or 21.4 | 240/2337 | 1.428 (1.171-1.742) | <0.001 | 1.316 (1.071-1.618) | 0.009 | 1.288 (1.037-1.600) | 0.022 |
| Cut points^*^: LDL-C 120.0 or 119.9 mg/dL; RC 21.5 or 21.4 mg/dL | | | | | | | | |
| <cut point | <cut point | 104/1478 | Reference | 1.0 | Reference | 1.0 | Reference | 1.0 |
|  | ≥cut point | 102/862 | 1.733 (1.313-2.286) | <0.001 | 1.579 (1.186-2.102) | 0.002 | 1.700 (1.263-2.288) | <0.001 |
| ≥cut point | <cut point | 75/872 | 1.279 (0.950-1.723) | 0.105 | 1.317 (0.977-1.776) | 0.071 | 1.423 (1.032-1.962) | 0.031 |
|  | ≥cut point | 138/1475 | 1.470 (1.135-1.903) | 0.003 | 1.393 (1.069-1.815) | 0.014 | 1.337 (1.005-1.778) | 0.046 |

Model 1: Adjusted for age, race, and sex. Model 2: Adjusted for model 1 covariates plus antihypertensive, BMI, CRP, drinking status, eGFR, FG, hypoglycemic medication use, SBP and smoking status. Model 3: Adjusted for model 2 covariates plus lipid-lowering medication use during follow-up. ^*^Cut points for model 1, and 2: LDL-C 120.0 mg/dL; RC 21.5 mg/dL; cut points for model 3: LDL-C 119.9 mg/dL; RC 21.4 mg/dL. AVC, aortic valve calcium; BMI, body mass index; CI, confidence interval; CRP, C-reactive protein; eGFR, estimate glomerular filtration rate; FG, fasting glucose; HR, hazard ratio; LDL-C, low-density lipoprotein cholesterol; RC, remnant cholesterol; SBP, systolic blood pressure.

**Table S7. Risk of AVC Progression Across LDL-C Versus RC Concordant/Discordant groups by LDL-C Clinical Cut Points (100 and 130 mg/dL) and Percentile Equivalents for RC Excluding Those on Lipid-Lowering Therapy at Baseline (Model 1 and Model 2, n=4210; Model 3, n=3746)**

| **LDL-C groups** | **RC groups** | **Events/No. at risk** | **Model 1**  **HR (95%CI)** | ***P* value** | **Model 2**  **HR (95%CI)** | ***P* value** | **Model 3**  **HR (95%CI)** | ***P* value** |
| --- | --- | --- | --- | --- | --- | --- | --- | --- |
| Cut points 1^*^: LDL-C 100 mg/dL; RC 16.6 mg/dL | | | | | | | | |
| <cut point | <cut point | 26/427 | Reference | 1.0 | Reference | 1.0 | Reference | 1.0 |
|  | ≥cut point | 61/579 | 1.592 (1.023-2.477) | 0.039 | 1.513 (0.969-2.363) | 0.069 | 1.604 (1.006-2.558) | 0.047 |
| ≥cut point | <cut point | 31/584 | 0.883 (0.542-1.437) | 0.616 | 0.933 (0.572-1.523) | 0.782 | 0.894 (0.529-1.512) | 0.676 |
|  | ≥cut point | 266/2650 | 1.602 (1.089-2.355) | 0.017 | 1.536 (1.040-2.268) | 0.031 | 1.557 (1.027-2.359) | 0.037 |
| Cut points 2^*^: LDL-C 130 mg/dL; RC 24.8 or 24.7 mg/Dl | | | | | | | | |
| <cut point | <cut point | 143/1945 | Reference | 1.0 | Reference | 1.0 | Reference | 1.0 |
|  | ≥cut point | 95/746 | 1.723 (1.335-2.223) | <0.001 | 1.568 (1.206-2.038) | 0.001 | 1.589 (1.210-2.088) | 0.001 |
| ≥cut point | <cut point | 71/745 | 1.385 (1.053-1.823) | 0.020 | 1.447 (1.096-1.912) | 0.009 | 1.475 (1.095-1.986) | 0.011 |
|  | ≥cut point | 75/804 | 1.551 (1.184-2.030) | 0.001 | 1.469 (1.115, 1.936) | 0.006 | 1.365 (1.015-1.837) | 0.040 |

Model 1: Adjusted for age, race, and sex. Model 2: Adjusted for model 1 covariates plus antihypertensive, BMI, CRP, drinking status, eGFR, FG, hypoglycemic medication use, SBP and smoking status. Model 3: Adjusted for model 2 covariates plus lipid-lowering medication use during follow-up.

^*^Cut points 1 for model 1, and 2: LDL-C 100 mg/dL; RC 16.6 mg/dL; cut points 2 for model 1, and 2: LDL-C 130 mg/dL; RC 24.8 mg/dL. Cut points 1 for model 3: LDL-C 100 mg/dL; RC 16.6 mg/dL; cut points 2 for model 3: LDL-C 130 mg/dL; RC 24.7 mg/dL. AVC, aortic valve calcium; BMI, body mass index; CI, confidence interval; CRP, C-reactive protein; eGFR, estimate glomerular filtration rate; FG, fasting glucose; HR, hazard ratio; LDL-C, low-density lipoprotein cholesterol; RC, remnant cholesterol; SBP, systolic blood pressure.

**
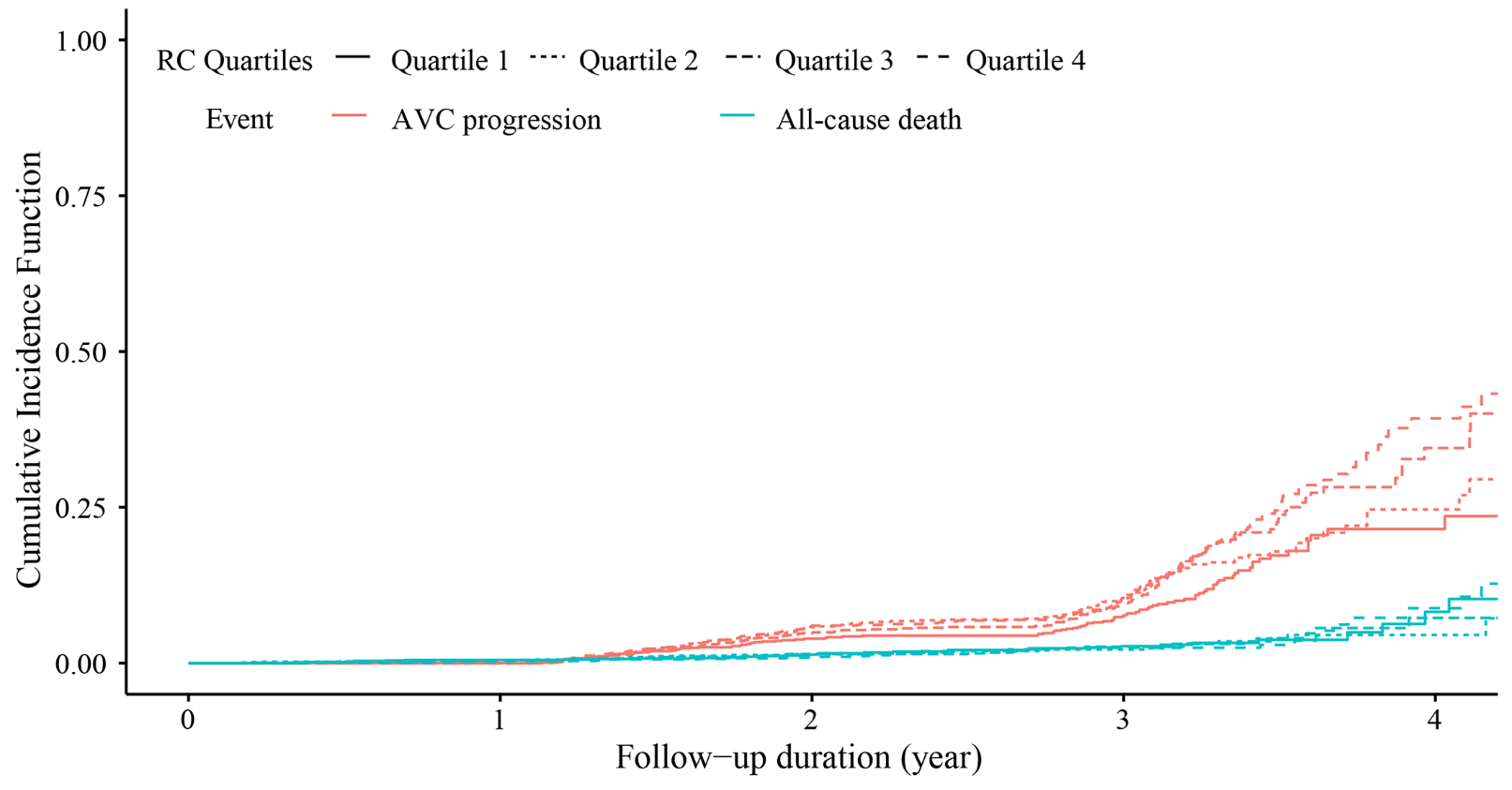
**

**Figure S1. Competing risk regression analysis.** Cumulative incidence function of follow-up years to AVC progression with all-cause death as a competing risk among different RC quartiles by using the Fine and Gray model. AVC, aortic valve calcium; RC, remnant cholesterol.
